# Lipome du corps calleux: à propos d'un cas avec revue de littérature

**DOI:** 10.11604/pamj.2015.21.245.4069

**Published:** 2015-08-06

**Authors:** Bouchra Zhari, Houda Mattiche, Hassan Boumdine, Touriya Amil, Hassan Ennouali

**Affiliations:** 1 Service d'Imagerie Médicale, Hôpital Militaire d'Instruction Mohamed V, Rabat, Maroc

**Keywords:** Lipome intracrânien, lipome du corps calleux, agénésie du corps calleux, intracranial lipoma, lipoma of the corpus callosum, agenesis of the corpus callosum

## Abstract

Le lipome du corps calleux est une malformation congénitale très rare, qui peut être associée à des degrés divers de dysgénésie du corps calleux. Son extension dans le ventricule latéral est encore plus rare. Il est souvent asymptomatique, mais peut se présenter par une épilepsie, hémiplégie, démence ou de simples céphalées. La tomodensitométrie et l'imagerie par résonance magnétique permettent facilement le diagnostic. Nous rapportons le cas d'une jeune fille de 14 ans, souffrant de simples céphalées, chez qui on découvre un lipome du corps calleux avec extension au ventricule latéral.

## Introduction

Les lipomes intracrâniens sont des lésions congénitales malformatives très rares, moins de 0,1% des tumeurs intracrâniennes [1]. Ils sont considérés comme des hétérotopies plutôt que des tumeurs, car ils sont histologiquement composés de cellules graisseuses normales, mais anatomiquement déplacées [2]. Ils se produisent principalement dans la région péri calleuse (45%) [3]. Les lipomes du corps calleux ne représentent toutefois que 5% des tumeurs calleuses [3]. Ils sont associés dans plus de la moitié des à des malformations congénitales telles que l′agénésie / dysgénésie du corps calleux [1]. La plupart des lipomes du corps calleux sont asymptomatiques et sont découverts fortuitement. Leur Pronostic et les symptômes dépendent de la malformation associée.

## Patient et observation

**Figure 1:**
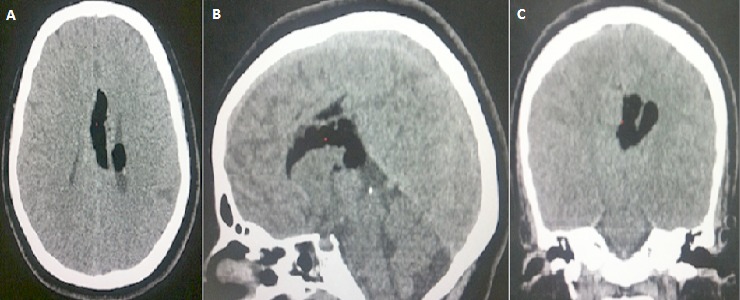
(A,B,C): TDM cérébrale en contraste spontané, en coupes axiale, coronale et sagittale, montrant: une formation de densité graisseuse de la ligne médiane, occupant la partie postérieure du corps calleux et s'allonge au corps, mesurant 16mm d’épaisseur, et s’étend au ventricule latéral gauche. Il s'y associe une dysgénésie du corps calleux

**Figure 2:**
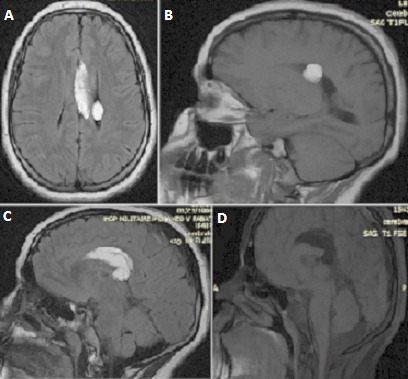
(A,B,C,D): IRM cérébrale, en Sp T1, T2 FLAIR, et saturation de graisse, en coupes axiale, coronale et sagittale, montrant: une formation de la ligne médiane, en hyper signal T1 et T2 FLAIR, s'effaçant après saturation de la graisse, siégeant au niveau du splénium, qui est dysgénésié, et s'allonge au dessus du corps calleux jusqu'au genou avec, qui est dysgénésié, avec extension dans le ventricule latéral gauche

Il s'agit d'une patiente de 14 ans sans antécédents pathologiques notables, qui rapporte des céphalées avec baisse de l'acuité visuelle, sans autres signes particuliers. L'examen neurologique était normal, et la patiente nous a été adressée pour la réalisation d'une TDM cérébrale. Le scanner a révélé (Figure 1): la présence d'une formation de densité graisseuse de la ligne médiane, occupant la partie postérieure du corps calleux et s'allonge au corps, mesurant 16 mm d’épaisseur, et s’étend au ventricule latéral gauche,où elle est étroitement appliquée au plexus choroïde, avecdysgénésie du corps calleux. On notait également la présence au niveau du rebord supérieur de quelque micro calcifications. Pour une meilleure caractérisation, un complément par une IRM cérébrale a été réalisée, et a montré (Figure 2) comme le scanner: une formationde la ligne médiane, qui apparaît en hyper signal T1 et T2 FLAIR, siégeant au niveau du splénium et s'allongeant au dessus du corps calleux jusqu'au genou. Cette formation présentait une extension dans le ventricule latéral gauche. Une séquence de suppression de la graisse a été réalisée, et la lésion a chuté de signal confirmant ainsi le diagnostic. Le diagnostic du lipome du corps calleux étendu au ventricule latéral a été retenu.

## Discussion

Les lipomes intracrâniens sont des lésions congénitales malformatives très rares, moins de 0,1% des tumeurs intracrâniennes. Ce sont des anomalies de différenciation du tissu mésenchymateux de la méninge primitive. Ils ont été décrits pour la première fois en 1818 par Meckel (lipome chiasmatique), et en 1856 par Rokitansky qui a décrit un lipome péri calleux avec agénésie du corps calleux [1].

Ils sont situés sur la ligne médiane dans 90% des cas, et le site le plus fréquent est la région dorsale péri calleuse [2]. Toutefois, bien qu'ils siègent dans 45% des cas au niveau du corps calleux, les lipomes du corps calleux ne représentent que 5% des tumeurs calleuses [3]. Ils sont souvent associés à d′autres anomalies de la différenciation des structures médianes, notamment à une hypogénésie ou agénésie du corps calleux, qui sont retrouvés dans 90% des lipomes antérieurs et dans 30% des lipomes postérieurs [1].

L′étiopathogénie précise des lipomes intracrâniens est encore un sujet de discussion. Historiquement, plusieurs théories concernant l'histogenèse de ces lésions ont été avancées, notamment: l′hypertrophie du tissu adipeux pré existant dans les méninges, la métaplasie du tissu conjonctif méningé, malformation hétérotopique d′origine dermique, et pseudo tumeur dérivée de la méninge primitive. Aujourd'hui, on admet que ces lipomes sont une anomalie de la différenciation de la méninge primitive persistante, qui se résorbe normalement entre la 8ème et la 10ème semaine de gestation [4]. Les autres anomalies liées à la présence des lipomes intracrâniens comprennent une agénésie du corps calleux (la plus courante), une agénésie du vermis cérébelleux, des tumeurs de l′hypophyse, des schwanomes acoustiques et d'autres lipomes intracrâniennes plus souvent situés dans le plexus choroïde des ventricules latéraux [4, 5].

Les lipomes de corps calleux sont morphologiquement classés en deux groupes [1]: les lipomes antérieurs (tubulonodulaires): ils sont arrondis ou lobulaires et mesurent généralement> 2cm d′épaisseur. Ils sont fréquemment associés à une hypogénésie / agénésie du corps calleux, des anomalies des lobes frontaux, calcifications, et / ou des anomalies oculaires. La variété tubulonodulaire peut s′étendre dans les plexus choroïdes des ventricules latéraux; lipomes postérieurs (curvilignes): ils sont minces et allongés le long de la marge du corps calleux, et mesurent habituellement < 1 cm d′épaisseur. Ils siègentplus en arrière sur le splénium et sont moins souvent associés à des anomalies corps calleux et/ou d′autres anomalies encéphaliques.

Ils sont souvent rencontrés chez la population pédiatrique etl'adulte jeune, sur la base d'une imagerie cérébrale, soit de façon fortuite, ou pour exploration des symptômes qu′ils occasionnent [4]. Actuellement, avec l′utilisation croissante de l′échographie prénatale, de plus en plus de cas sont détectés, et ce à partir de la 26 semaine de gestation [1]. Les lipomes isolés du corps calleux sont asymptomatiques. Les manifestations cliniques comme des crises convulsives, des troubles mentaux, une hémiparésie et des céphalées sont souvent secondaires aux anomalies concomitantes du tissu nerveux. L′épilepsie est l'un des symptômes les plus fréquents: lorsqu′elle est présente, elle apparaît avant l’âge de 15 ans, et elle est souvent partielle et sévère. Ils peuvent aussi causer une hydrocéphalie obstructive [1].

L'imagerie est le moyen principal dans le diagnostic des lipomes du corps calleux. Les radiographies du crâne peuvent montrer des calcifications curvilignes surtout dans la variété tubulonodulaire. Sur la tomodensitométrie, ces lipomes apparaissent comme des masses de densité graisseuses (-80 à-110 UH), qui peuvent contenir des calcifications périphériques (la variété tubulonodulaire peut présenter une calcification curviligne périphérique parfois appelé «bracket sign » sur les images reconstruites coronales) [1]. L′IRM est ainsi l′examen de choix non seulement pour caractériser l'extension du lipome, mais aussi à la recherche de l′agénésie / dysgénésie du corps calleux fréquemment associées. Il n′est pas surprenant de ces masses suivent l′intensité du signal de la graisse sur toutes les séquences: ainsi elles apparaissent en hyper T1 et T2, avec chute du signal sur les séquences de FATSAT.

En général il existe peu de diagnostic différentiel à évoquer devant ces masses graisseuses du corps calleux, à savoir:les kystes et les tératomes dermoïdes, une faux du cerveau « graisseuse »: en particulier devant le type curviligne, ou une rare transformation lipomateuse de certaines tumeurs: TNEP, épendymome, gliome [1].

Aucun traitement spécifique n′est habituellement exigé: le traitement anticonvulsivant est la modalité de choix dans les lipomes symptomatiques, et la chirurgie est rarement indiquée en raison de la forte vascularisation et l′adhésion de la lésion au parenchyme environnant [1].

## Conclusion

Le lipome du corps calleux est une anomalie très rare, qui peut être associée à des degrés divers de dysgénésie du corps calleux. Son extension dans le ventricule latéral est encore plus rare. Dans la plupart des cas ils sont asymptomatiques et sont découverts fortuitement. Leur Pronostic et les symptômes dépendent de la malformation associée.
